# Medicine, healthcare and the AI act: gaps, challenges and future implications

**DOI:** 10.1093/ehjdh/ztaf041

**Published:** 2025-04-23

**Authors:** Emmanouil P Vardas, Maria Marketou, Panos E Vardas

**Affiliations:** Department of Cardiology, Athens General Hospital G. Gennimatas, Athens, Greece; Department of Cardiology, Heraklion University General Hospital, Heraklion, Crete, Greece; Medical School, University of Crete, Voutes, Heraklion, 70013 Crete, Greece; Medical School, University of Crete, Voutes, Heraklion, 70013 Crete, Greece; Biomedical Research Foundation, Academy of Athens, Soranou Efesiou 4, 115 27 Athens, Greece; Heart Sector, Hygeia Hospitals Group (HHG), 5, Erithrou Stavrou, Marousi, 15123 Athens, Greece

**Keywords:** Artificial, Intelligence, AI Act, Cardiovascular, Quality management systems

## Abstract

The European Union's Artificial Intelligence Act (AI Act), published in July 2024, is a pioneering horizontal regulatory framework aimed at ensuring the ethical and safe integration of AI technologies across sectors, including healthcare. While it offers the potential to improve patient care and drive innovation, it also presents challenges for healthcare providers, such as identifying high-risk applications, ensuring transparency in algorithms, and mitigating data bias. However, there are several challenges in its implementation. These include unclear guidance for certain technologies, the need to ensure fairness for diverse patient populations, effective monitoring of AI performance after deployment, and clarifying responsibility in cases of errors. Additionally, varying levels of resources among EU countries may lead to inconsistent implementation of the regulations. This article explores the core elements of the AI Act and its relevance to cardiology and identifies key gaps and unanswered questions that need to be addressed to effectively advance AI-driven medical practices.

## Graphical Abstract

**Graphical Abstract:**
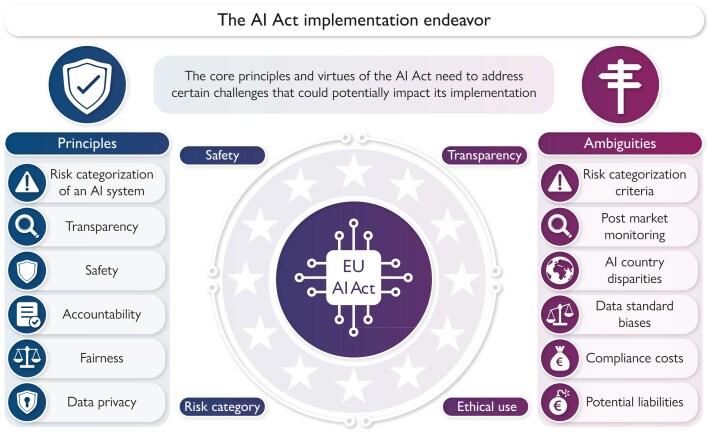

## Introduction

Artificial intelligence is progressing steadily, offering the potential to enhance medicine and, by extension, care through validated solutions for diagnosis, treatment, and personalized care.^1–4^ Undoubtedly, these significant advancements come with multiple challenges, necessitating a robust governance framework from the outset to ensure ethical standards, safety, fairness, and accountability.^5,6^

In July 2024, the Artificial Intelligence Act, a landmark regulatory framework, was published in the official journal of the European Union.^7^ This framework is poised to govern, horizontally, the development and deployment of AI systems within the EU, aiming to create a structured and consistent regulatory environment for AI systems. Notably, the AI Act is the first global regulation specifically targeting AI, setting a precedent for international AI governance.

Although the Medical Devices Regulation (MDR) primarily provides regulation for medical devices, the AI Act, in certain cases, could also be combined, playing a significant role.

This development could be seen as a significant opportunity to further improve quality indicators in healthcare by combining innovation with strict adherence to safety and ethical standards for medical devices. However, a set of perplexities and challenges renders this landscape rather uncertain, particularly for the providers and deployers of these devices and systems.

Both the AI Act and MDR focus on safety and transparency but approach it from different angles tailored to their specific domains, (*Table 1*).

**Table 1:** Comparative analysis of overlapping regulatory challenges under the AI act and MDR

Topic	Description
Conformity assessment	Assessment processes differ, creating ambiguity in compliance requirements for AI-based devices.
Risk classification systems	Variations in how risks are classified under AI Act and MDR can lead to regulatory discrepancies.
Data protection and privacy	Overlapping requirements for data handling could complicate compliance for device manufacturers.
Clinical evaluation and validation	Different standards for AI validation and medical device clinical trials challenge integration.
Post-market surveillance	Diverse expectations for ongoing monitoring under each regulation increase operational complexity.
Transparency and explainability	Both regulations demand transparency, but criteria and extent differ, affecting implementation.
Regulatory scope and coverage	Discrepancies in what devices are covered under each law can lead to enforcement challenges.
Software as a medical device (software as a medical device)	Specific issues for software as a medical device under both frameworks need harmonisation to avoid regulatory conflicts

The AI Act is broader, encompassing all AI applications with particular emphasis on high-risk scenarios, while the MDR is specifically tuned to the lifecycle of medical devices.^8^ Furthermore, the introduction of the European Health Data Space (EHDS) is set to play a pivotal role in supporting these regulations by ensuring the availability of large-scale health data, which could improve compliance, enhance development and evaluation.^9^

With the presentation of the aforementioned regulatory nexus of digital medical devices (DMD) in EU countries, we find it necessary to clarify, albeit very briefly, the roles and responsibilities of those authorized to issue regulatory rules and their implementation, namely regulators, and Notified Bodies (NBs).

NBs under the MDR are currently authorized to assess medical devices for compliance and issue certificates of conformity where required. The Ce marking, however, is placed by the manufacturer.^10^ The proposed AI Act is expected to extend similar responsibilities to high-risk AI systems, establishing a framework where NBs will assess AI products against set EU standards.

By intertwining these frameworks with the capabilities of the EHDS, Europe is positioning itself at the forefront of regulating advanced technologies in healthcare, ensuring that innovations deliver on their promise of improving patient outcomes while safeguarding fundamental rights and ethics.

This article focuses on key aspects of the AI Act in medicine and healthcare, acknowledging its significance while highlighting critical gaps, challenges, and uncertainties within the EU regulatory nexus. This nexus must harmoniously implement EU regulations to safeguard patient safety and device effectiveness without hindering technological progress and innovation.

### Core principles of the AI act

The robust regulatory framework is grounded in several core principles designed to ensure the safe and ethical use of artificial intelligence across diverse sectors, including healthcare. The implementation of the EU AI Act in member states introduces critical legal and ethical prerequisites for medical devices that utilize artificial intelligence. The Act categorizes AI systems based on risk, with medical devices generally classified as high-risk, necessitating strict compliance with safety and transparency standards. Manufacturers are required to establish comprehensive risk management and quality management systems (QMS) that align with existing medical device regulations, such as the MDR and IVDR.

Ethical considerations are paramount, ensuring that AI systems uphold fundamental rights, including data protection and patient safety. Additionally, ongoing post-market monitoring and incident reporting are mandated to maintain accountability and enhance trust in AI-driven healthcare solutions, reflecting a commitment to both innovation and public welfare in the medical sector.^6–8,11^

### Risk-based categorisation of AI systems in the AI act

The regulatory framework outlined in the Act is a risk-based classification system categorising AI tools into four tiers: unacceptable, high, limited, and minimal risk. Unacceptable risk AI systems, such as those used for social scoring or exploiting vulnerabilities, are outright banned. These applications are deemed incompatible with fundamental human rights and public safety.

High-risk AI applications, particularly in healthcare, such as diagnostic tools, AI-assisted surgeries, clinical decision support software, and patient monitoring systems, are subject to stringent regulations. These include rigorous pre-market conformity assessments and continuous post-market surveillance to ensure they meet high standards of safety, accuracy, and transparency. The regulatory framework mandates strict oversight to safeguard public health and ensure the efficacy and reliability of these critical technologies.

Limited-risk systems, like chatbots, require basic transparency obligations, such as informing users they are interacting with AI. Minimal or no-risk systems, such as spam filters, are exempt from stringent oversight, allowing for streamlined innovation.

For healthcare, this risk-based framework is critical. It ensures that high-risk systems, which directly impact patient outcomes, are thoroughly vetted, while low-risk innovations can proceed with minimal regulatory burdens. This proportional approach safeguards trust and safety while fostering advancement and innovation in AI technology.

Manufacturers of regulated digital products, such as medical devices, must adhere to QMS under the EU MDR/IVDR, typically following ISO/IEC 13485 standards and incorporating AI-specific requirements into their existing QMS as outlined in Article 17.^12^ Additionally, AI medical products should ensure ongoing compliance through technical documentation, design validation, performance testing, and risk management. Developers of AI medical devices must comply with both the EU MDR and the AI Act regulations, which often contain overlapping requirements, such as risk assessment, QMS, technical files, and post-marketing surveillance. Differing interpretations of similar requirements may cause confusion, especially for small and medium-sized enterprises, which may find it challenging to balance regulatory demands with their focus on engineering, quality, and product development.^6,13,14^

### Focus on safety, transparency

Corresponding to what was previously stated, the AI Act emphasizes safety, transparency, and accountability as essential pillars for integrating artificial intelligence into critical sectors, including healthcare. These principles are designed to ensure that AI systems operate reliably, ethically, and with minimal risk to users and society.

**Table 2:** Challenges and queries in the AI act applications for medical software

Critical areas	Description
Risk categorisation	Criteria for classifying AI risk levels are vague. Clarity is needed for borderline cases.
Data standards	Lack of standardisation. Several difficulties in rare diseases.
Transparency and interpretability	No clear standards. Concerns over ‘black-box’ models.
Post-market monitoring	Limitations in the ongoing evaluation. Challenges in real-world feedback.
Liability and accountability	Unclear allocation of responsibility among manufacturers and healthcare providers in case of AI system failures.
Harmonisation across the EU	Disparities in digital infrastructure and healthcare systems among EU nations may hinder consistent implementation.
Human oversight	Lack of defined qualifications or training requirements for individuals overseeing high-risk AI systems.

Safety is a cornerstone of the Act, requiring AI systems to undergo rigorous testing to prevent harm. For high-risk applications in healthcare, such as diagnostic algorithms or AI-powered surgical tools, safety protocols include pre-market evaluations, continuous monitoring, and risk management strategies. These measures are critical in preventing malfunctions or biases that could compromise patient outcomes, fostering confidence in AI among clinicians and patients alike.

Algorithmic bias occurs when the design of the algorithm produces unfair outcomes; for example, an AI tool predicting skin conditions may perform poorly on darker skin tones if such data were under-represented during training.^15,16^ In cardiology, an AI-based arrhythmia detection tool might fail to recognize patterns in patients with rare cardiac conditions if these cases are insufficiently represented. Data bias arises from imbalanced datasets, such as a cardiac risk model trained predominantly on middle-aged men, leading to underperformance in women or younger individuals. Selection bias can occur if wearable device data is collected only from healthy, tech-savvy users, reducing its effectiveness for older or multi-morbid patients. Automation bias may result in clinicians blindly accepting AI-driven ECG interpretations, potentially missing subtle but clinically significant anomalies.

Mitigating these biases through diverse datasets and continuous model validation is critical to ensure fairness and accuracy in healthcare AI, particularly in high-stakes fields like cardiology.

Transparency ensures that AI systems are understandable and their decision-making processes are interpretable. Developers must document the design, intended purpose, and potential limitations of their AI systems. For clinicians, this means having access to clear, actionable information about how the AI reaches its conclusions, enabling them to make informed decisions. Transparency also extends to informing patients when AI is used in their care, promoting trust and informed consent.

By prioritising these principles, the AI Act provides a framework for safe, effective, and trustworthy AI integration in healthcare.

### Mandatory conformity assessments for high-risk AI systems

In the European Union, high-risk AI-enabled medical devices are regulated by both the Medical Device Regulation (MDR, 2017) and the AI Act (2024). These frameworks aim to ensure safety, efficacy, transparency, and ethical use of such technologies. However, the responsibility for the mandatory conformity assessment for these devices is distributed depending on the focus of each regulation.^7,8^

The MDR (2017) governs the safety and performance of medical devices, including those with AI components. It requires that high-risk devices undergo conformity assessments conducted by NBs, focusing on clinical evaluation, risk management, and post-marker surveillance. These bodies ensure compliance with essential health and safety requirements.^8^

On the other hand, the AI Act (2024) establishes a framework for trustworthy AI systems. For high-risk AI applications, including those integrated into medical devices, it mandates conformity assessments addressing AI-specific risks like transparency, bias and robustness. This regulation designates NBs or equivalent authorities to evaluate AI-related risks and ethical standards.^7,10^

While both frameworks overlap, their scopes differ: the MDR emphasizes medical safety and device efficacy, while the AI Act addresses algorithmic transparency and ethical use. In practice, the NBs under MDR primarily handle the conformity assessment for high-risk AI-enabled devices, incorporating AI Act requirements where applicable. This ensures a comprehensive evaluation of both evaluation and AI-specific risks.

Pre—market evaluation is a critical step in the regulatory process, ensuring that high-risk AI-enabled medical devices meet the required safety and performance standards before entering the market.^6,7,17^ Under the MDR, NBs are tasked with rigorous pre-market conformity assessments, which include evaluating clinical evidence, risk management processes, and device efficacy. These assessments also integrate AI-specific evaluations under the AI Act to verify algorithmic robustness, bias mitigation and transparency.

Post-market monitoring ensures that AI-enabled medical devices remain safe and effective throughout their lifecycle. While both the MDR and the AI Act mandate continuous oversight, the MDR assigns primary responsibility to manufacturers for implementing surveillance systems, including safety updates, real-world performance evaluations, and corrective actions. The AI Act complements this by emphasising ongoing AI performance assessments to enhance transparency and mitigate algorithmic risks. Ultimately, manufacturers bear the main responsibility under the MDR’s framework, with the AI Act reinforcing AI-specific accountability.

The AI Act envisages that there shall be no duplications, and as such, the NBs that are competent to assess DMD can also be designated as NBs in accordance with the AI Act, to enable a joint Ce marking conformity assessment and technical documentation claiming compliance with MDR and the AI Act.

In practice, however, such combined conformity assessment may not always be easy for a number of different reasons, in several states, as there is no unified European regulatory institute to harmonize the implementation of the aforementioned regulations.

### Ethical use of data and privacy protections in the AI act

The AI Act emphasizes ethical data use and robust privacy protections, particularly in high-stakes sectors like healthcare. Central to this principle is the requirement for high-quality, diverse, and unbiased datasets to train AI systems. This ensures that algorithms function equitably, avoiding biases that could disproportionately affect specific patient groups.

Privacy protections align closely with the General Data Protection Regulation (GDPR), reinforcing strict compliance when processing sensitive personal data, such as health information.^18–20^ In AI development, training data teaches the model to identify patterns and make predictions. Validation data optimizes the model during development by fine-tuning parameters, while testing data evaluates its performance on unseen cases. In a clinical setting, operational data refers to real-world patient inputs, distinct from development datasets, as it drives the AI tool's decision-making and outcomes. Developers must ensure the protection of patient identities throughout the AI lifecycle. This involves robust anonymization, encryption, and compliance with legal frameworks like GDPR to safeguard sensitive information. Upholding privacy is vital for ethical AI use and maintaining trust in clinical applications.

For researchers, these principles demand rigorous attention to data sourcing, quality, and security during algorithm development. Clinicians, as end-users, must ensure the AI systems they deploy adhere to these standards. By prioritising ethical data use and privacy, the AI Act builds trust in AI while safeguarding patient rights and data integrity.

### Gaps and queries in the AI act

Undoubtedly, the AI Act has elevated the EU as a global leader in AI governance, ensuring that AI technologies are developed and used in a manner that is safe, ethical, and respects fundamental rights. However, it is evident, as has become apparent immediately after the publication of this regulatory framework that particularly in the case of medicine and healthcare, there will be difficulties in its implementation^21^ and significant needs to clarify uncertainties and gaps, which are particularly important in the high-stakes field of healthcare professional services, (*Table 2*).

The EHDS is poised to play a role in supporting the implementation of the AI Act within healthcare.^9,22^ By establishing a standardized framework for data usage and privacy, EHDS can provide the necessary infrastructure to ensure that AI applications comply with EU regulations. This synergy could streamline the integration of AI technologies by clarifying data access, portability and interoperability across member states. Furthermore, EHDS’s emphasis on secure and seamless data exchange will facilitate the development of AI systems that are both innovative and compliant with EU standards, thereby accelerating their adoption and effectiveness in clinical settings.

### Risk categorisation: a framework in evolution

The AI Act classifies AI systems into four categories based on risk—an essential and logical approach. However, this also presents several challenges, as the criteria for categorising risk are often vague, particularly for emerging technologies that defy traditional definitions. The key question here is how regulators and NBs will address borderline cases and whether there is a mechanism to periodically reclassify available systems as technologies and their applications continually evolve.

Unfortunately, for the European context, the situation appears even more challenging and complex, as the existing regulatory framework for DMDs will become progressively more demanding.

It is evident that, in this new regulatory landscape, where AI-enabled medical devices fall under the jurisdiction of both regulatory frameworks—the AI Act (2024) and the MDR (2017)—a harmonized assessment is required.

The solution would be provided by well-empowered common national NBs, or an EU-coordinated regulatory hub for developing uniform evaluation criteria, and the harmonized application of existing regulations.

At present, it is evident that the AI Act needs to be supported with a clear definition of the boundaries between risk categories to reduce uncertainties for all stakeholders—patients, healthcare professionals, and developers alike.

### Data standards and the problem of bias

The AI Act highlights the need for robust, unbiased, and representative datasets for training AI systems. This is especially critical in healthcare, where biased algorithms can exacerbate health disparities.

However, for several reasons, these standards pose significant challenges, particularly in Europe, where there is a notable lack of data standardisation. Additionally, datasets for rare diseases are often geographically limited, making it difficult to meet the Act's representativeness criteria.

It is evident that ongoing monitoring of bias mitigation efforts is crucial. Unfortunately, at present, this need remains largely unaddressed. Establishing a framework for auditing datasets and algorithms over time could help bridge this gap.

By establishing a standardized framework for data usage and privacy, EHDS can provide the necessary infrastructure to ensure that AI applications comply with EU regulations. This synergy could streamline the integration of AI technologies by clarifying data access, portability, and interoperability across member states.

Furthermore, EHDS’s emphasis on secure and seamless data exchange will facilitate the development of AI systems that are both innovative and compliant with EU standards, thereby accelerating their adoption and effectiveness in clinical settings.

One of the AI Act's key cornerstones, particularly for high-risk AI systems, is openness, transparency, and the interpretability of the developed software. This principle is especially critical in medicine, where clinicians must understand, at least to some extent, how the algorithm arrives at its recommendations.

At this point, it is worth noting that the EU Act does not provide concrete standards for what constitutes ‘acceptable’ transparency, as defined in the text, particularly in fields of special significance like medicine. Based on this, the pressing question arises whether the EU will soon supply detailed guidelines on interpretability, at least for specific sectors like medicine, and how ‘black-box’ models will be regulated in these domains.

With respect to the applicable requirements and prerequisites of existing regulations, we could speculate here that the requirements for access by clinicians to the algorithms of AI-enabled medical devices, and the so-called ‘algorithmic transparency’ as it is currently recognized, may gradually be deprioritized over time. Instead, the accuracy of closed-loop systems may take precedence, primarily focusing on the precision and speed of outcomes.

This shift towards prioritising the accuracy of AI devices may open up new possibilities for the role of physicians. With some ‘in the loop’, actively intervening, and others ‘on the loop’, monitoring decisions, this change could enhance the collaboration between technological efficiency and clinical expertise.

### Post-market monitoring as an imperative need

An analysis of the AI Act reveals its heavy focus on pre-market compliance, emphasising requirements for testing, validation, and certification. In contrast, the post-market evaluation of AI systems and models—where real-world challenges often surface—receives less attention.

It is evident that the Act does not propose a detailed framework for ongoing monitoring, reporting adverse events, or adapting systems based on new data. However, in the context of medicine and healthcare, as evidenced by the history of high-risk devices, post-market surveillance is critical for evaluating outcomes and detecting rare but serious failures.

A centralized reporting system to track and respond to AI-related incidents will likely be necessary. This system would enable the creation of feedback loops between clinicians, regulators, and developers. Without robust post-market mechanisms, the Act risks establishing a regulatory framework that falls short in real-world application compared with its theoretical intent.

Το ensure the AI Act meets its intended real-world applications, robust post-market mechanisms are essential, complementing the initial oversight provided by NBs. While NBs play a pivotal role in assessing compliance before market entry, the dynamic nature of AI systems necessitates continuous evaluation after deployment.

Key challenges include performance drift, where models degrade over time due to shifts in data distributors and self-learning systems, which evolve autonomously, potentially diverging from their original validation. Addressing these requires a framework for ongoing monitoring, periodic revalidation and transparent reporting mechanisms.

Establishing clear protocols for managing drift, retaining models and auditing adaptive systems will help align their real-world performance with regulatory standards. Such measures are crucial for maintaining trust, safety and clinical relevance in dynamic healthcare environments.

### Liability and accountability in the AI act era: a complex landscape

The AI Act addresses mostly accountability and liability in AI-enabled medical devices to ensure responsible deployment.^7,23–25^

The allocation of responsibility for AI system failures is a complex issue, particularly in collaborative environments such as healthcare.^24^

With the broader adoption of AI systems, especially in healthcare, legal experts, users, and developers will need to collaborate to define the nuances of potential liabilities.

Currently, the AI Act places the primary burden on providers and distributors, leaving critical questions unanswered. Specifically, the Act does not clearly define the roles of other stakeholders, such as hospitals or clinicians, in ensuring compliance and addressing failures.

Additionally, it remains unclear how liability will be distributed when multiple parties are involved, such as in the case of a diagnostic medical tool producing an error, with developers, healthcare providers, and clinicians all participating in its use. This lack of clarity could deter innovation, as stakeholders may hesitate to adopt technologies without clear liability protections.

Last but not least, even in brief, we must recognize the importance of ensuring coverage, as a central matter related to the clinical usage of AI-enabled medical devices. While the EU AI Act does not explicitly detail provisions on this aspect, it establishes a regulatory framework that significantly impacts how insurers must approach the risks associated with medical devices. Insurers will need to adapt their practices to align with both the new AI regulations and existing insurance laws to effectively manage related challenges.

### Harmonising AI regulations across the EU: addressing disparities

The AI Act aspires to create a unified regulatory framework across all EU member states. However, disparities in digital infrastructure and healthcare systems present significant challenges, potentially undermining the Act's successful implementation.

Moreover, possible heterogeneities in staffing, quality, and effectiveness of the NBs among EU states could exacerbate the challenges posed by existing disparities, potentially making the uniform application of the Act across the European Union problematic.

Furthermore, it is critical to highlight that the Act does not address how these disparities will impact implementation or how fragmentation between states will be avoided. More developed member states with advanced digital ecosystems may adapt quickly, while others lag behind.

This raises the question of whether the EU will need to provide additional support to under-resourced regions to ensure equitable adoption. Harmonising AI Act implementation across all 27 EU member states is a fundamental priority, particularly in cross-border healthcare, where inconsistencies between MDR, AI Act and EHDS or any inconsistent implementation of the regulations could hinder data sharing and collaborative AI projects.

### Human oversight as a distant prospect for AI accountability

Under the EU regulatory framework, human oversight is required for high-risk AI systems to ensure accountability. While this requirement appears logical and essential, its practical implementation remains ambiguous.

Specifically, the Act does not define the qualifications or training necessary for individuals overseeing AI systems, particularly for models and software specialized in medicine. These observations underscore the need for specialized training, standardized certifications, or detailed guidelines to enable proper human oversight, balancing AI efficiency with reasonable risk assessment.

In conclusion, the AI Act represents a significant step towards establishing a regulatory framework that ensures the safe, ethical, and transparent development of AI technologies, particularly in high-risk sectors like healthcare. Its risk-based classification system is designed to ensure that AI systems impacting patient outcomes undergo rigorous testing, evaluation, and ongoing monitoring. By prioritising safety, transparency, and accountability, the Act fosters trust and confidence in AI applications, which is critical for their adoption in healthcare.

However, several gaps remain, particularly in the areas of risk categorisation, data standards, and post-market monitoring. The Act’s provisions for data privacy, algorithmic fairness, and interpretability are essential but still require clearer guidelines, especially for complex medical AI tools. Additionally, the lack of clarity around liability and human oversight, combined with disparities in digital infrastructure across EU member states, presents challenges to consistent implementation.

Addressing these gaps through ongoing updates and more detailed regulations will be crucial to ensure that the AI Act effectively supports innovation while safeguarding patient safety and equity. As AI technologies continue to evolve, continuous dialogue between regulators, healthcare professionals, and developers will be necessary to adapt the regulatory landscape to emerging challenges and opportunities in AI-driven healthcare.

## Data Availability

The data underlying this manuscript are derived from publicly available sources. All articles can be accessed through the references listed in the manuscript.

## Lead author biography

Emmanuel P. Vardas is a Greek physician and trainee in electrophysiology and cardiology. Over the past 6 years, Dr Vardas has developed a strong interest in Digital Health and Artificial Intelligence. His ongoing doctoral research focuses on the diagnostic role of mRNAs as biomarkers for atrial fibrillation recurrences in pulmonary vein isolation patients within specific atrial fibrillation subgroups.
